# Integrated analysis of plasma proteome and cortex single-cell transcriptome reveals the novel biomarkers during cortical aging

**DOI:** 10.3389/fnagi.2023.1063861

**Published:** 2023-07-19

**Authors:** Rui-Ze Niu, Wan-Qing Feng, Qing-Shan Yu, Lan-Lan Shi, Qing-Min Qin, Jia Liu

**Affiliations:** Laboratory Animal Department, Kunming Medical University, Kunming, China

**Keywords:** brain aging, single-nuclear transcriptome sequencing, proteome, biomarker, plasma

## Abstract

**Background:**

With the increase of age, multiple physiological functions of people begin gradually degenerating. Regardless of natural aging or pathological aging, the decline in cognitive function is one of the most obvious features in the process of brain aging. Brain aging is a key factor for several neuropsychiatric disorders and for most neurodegenerative diseases characterized by onset typically occurring late in life and with worsening of symptoms over time. Therefore, the early prevention and intervention of aging progression are particularly important. Since there is no unified conclusion about the plasma diagnostic biomarkers of brain aging, this paper innovatively employed the combined multi-omics analysis to delineate the plasma markers of brain aging.

**Methods:**

In order to search for specific aging markers in plasma during cerebral cortex aging, we used multi-omics analysis to screen out differential genes/proteins by integrating two prefrontal cortex (PFC) single-nucleus transcriptome sequencing (snRNA-seq) datasets and one plasma proteome sequencing datasets. Then plasma samples were collected from 20 young people and 20 elder people to verify the selected differential genes/proteins with ELISA assay.

**Results:**

We first integrated snRNA-seq data of the post-mortem human PFC and generated profiles of 65,064 nuclei from 14 subjects across adult (44–58 years), early-aging (69–79 years), and late-aging (85–94 years) stages. Seven major cell types were classified based on established markers, including oligodendrocyte, excitatory neurons, oligodendrocyte progenitor cells, astrocytes, microglia, inhibitory neurons, and endotheliocytes. A total of 93 cell-specific genes were identified to be significantly associated with age. Afterward, plasma proteomics data from 2,925 plasma proteins across 4,263 young adults to nonagenarians (18–95 years old) were combined with the outcomes from snRNA-seq data to obtain 12 differential genes/proteins (GPC5, CA10, DGKB, ST6GALNAC5, DSCAM, IL1RAPL2, TMEM132C, VCAN, APOE, PYH1R, CNTN2, SPOCK3). Finally, we verified the 12 differential genes by ELISA and found that the expression trends of five biomarkers (DSCAM, CNTN2, IL1RAPL2, CA10, GPC5) were correlated with brain aging.

**Conclusion:**

Five differentially expressed proteins (DSCAM, CNTN2, IL1RAPL2, CA10, GPC5) can be considered as one of the screening indicators of brain aging, and provide a scientific basis for clinical diagnosis and intervention.

## 1. Introduction

Nowadays, human life expectancy in general has increased dramatically, and the phenomenon of population aging becomes one of the most significant public health problems worldwide. It is well known that the function of the brain will gradually decline during the aging process, mainly manifested by barriers in learning and memory, attention, decision-making speed, sensory perception and motor coordination (Alexander et al., 2012). Mendonca et al. (2017) suggested that the decline of brain function roughly parallels the time course of decline in other organ functions and accelerates significantly after age 50. The current aging trend greatly depresses the average quality of life of the elderly, but also increases the pressure on family and society for providing for the aged. Brain aging is mainly divided into normal aging and pathological aging, and pathological brain aging represents as one leading factor of increasing incidence of neurodegenerative diseases including Alzheimer’s disease (AD), Parkinson’s disease (PD), etc (Isaev et al., 2019). However, there is currently no substantial diagnostic and therapeutic methods for the prevention of brain aging and the relief of symptoms.

Single-cell/nucleus RNA sequencing (scRNA-seq/snRNA-seq) techniques have unraveled the transcriptional alterations underlying the heterogeneous process of aging at individual cell-type-specific resolution in multiple organs (Zhang et al., 2021). Compared with scRNA-seq, snRNA-seq can directly extract nuclei from frozen tissues to perform sequencing (Whytock et al., 2022). Moreover, snRNA-seq provides unique advantages in analyzing tissues difficult to be dissociated, such as the neuron-rich brain tissues, thus reducing the bias in cellular capture and the accompanied transcriptional artifacts (Zhang et al., 2021). At present, many scholars are more inclined to use snRNA-seq for scientific research on brain tissue (Preissl et al., 2018; Zhong et al., 2020; Hao et al., 2022). The plasma proteome is a research analysis method that focuses on the study of proteins in blood. The plasma proteome profile changes with age throughout the human life cycle, as does the aging of organs (Lehallier et al., 2019), and has played a significant role in the study and exploration of neurological-related diseases (He et al., 2021; Sorek et al., 2021), cardiovascular diseases (Roth et al., 2020; Wang et al., 2021), tumors (Riemondy et al., 2022), and ocular retinopathy (Menon et al., 2019). This method provides new ideas for the diagnosis, prevention and subsequent research of various diseases by utilizing the differential expression of plasma proteome in different physiological states and disease states, and also shows some prospective and application prospects in clinical practice.

In this study, we have constructed the transcriptome profile of PFC aging by integrating two datasets from two independent snRNA cohort study (Brenner et al., 2020; Lau et al., 2020), and explored new biomarkers about the PFC aging through comprehensive integrated analysis of plasma proteome and cortical single-cell transcriptome (Liu and Trapnell, 2016). We finally identified five candidate proteins associated with brain aging in plasma and verified them as potential biomarkers of brain aging by ELISA test. This study provides novel diagnostic basis and research ideas for the prevention and treatment of a variety of neurodegenerative diseases related to aging in the future.

## 2. Materials and methods

### 2.1. Datasets and subjects

Two snRNA-seq datasets (GSE141552 and GSE157827) were obtained from Gene Expression Omnibus (GEO).^1^ These datasets were obtained using the 10X Genomics platform and NovaSeq 6000 sequencing platform (Brenner et al., 2020; Lau et al., 2020). The data of plasma proteome were obtained from the “Aging Plasma Proteome^2^” (Lehallier et al., 2019).

### 2.2. SnRNA-seq analysis

#### 2.2.1. Preprocessing, quality control, and data integration

The gene barcode matrices for each sample were loaded into the R using the Read 10X function in the Seurat R package (Nagy et al., 2020). The Seurat Object, corresponding to each sample was created using the Create Seurat Object function with the input gene barcode matrix provided as the raw data. The datasets were integrated using the method of Stuart et al. (2019). Data quality was controlled prior to integration. The number of genes per sample, unique molecular identity counts, and the percentage of mitochondrial genes were controlled. To exclude potential dead cells and cell debris from the dataset, we filtered out nuclei with ≤200 genes, ≥7500 unique molecular identifiers, or ≥5% mitochondrial genes. In total, 65,064 high-quality nuclei were obtained for subsequent analyses. For the integration analysis, the highly variable features of each sample were identified using the *FindVariableFeatures* function with selection.method = vst, nfeatures = 2000. To integrate all the samples, the *FindIntegrationAnchors* function was used to anchor the features of the samples with dims = 1:30. All samples were integrated using the *IntegrateData* function with the parameter dims = 1:30.

#### 2.2.2. Data dimension reduction and clustering analysis

Subsequently, we scaled the expression matrix and performed a linear dimension reduction using the *RunPCA* function with the parameter npcs = 50. The *P*-value distribution of each major component was visualized using the *JackStrawPlot* function and selected to perform graph-based clustering using the first 30 principal components. We performed K-nearest neighbor (KNN) clustering using the *FindClusters* function with the parameter resolution = 1 and UMAP clustering using the *RunUMAP* function with the parameter dims = 1:30, which initially yielded 22 cell clusters. Differentially expressed genes (DEGs) in each cell cluster were identified by the Wilcoxon rank-sum test using the *FindAllMarkers* function with the parameters logfc.threshold = 0.25 and test.use = wilcox. We then assigned a cell-type identity to each cell cluster according to the expression of known cell-type markers and identified additional cell type-specific marker genes by the Wilcoxon rank-sum test using the *FindAllMarkers* function with the parameters logfc.threshold = 0.25 and test.use = wilcox. For cell-type markers, the level of statistical significance was set at an adjusted *P*-value < 0.05.

#### 2.2.3. Examination of cell type-specific transcriptomic changes

To examine cell type-specific transcriptome changes in the cerebral cortex of the aging group, we used the FindMarkers function and parameters logFc.threshold = 0.25 and test.use = Wilcox by Wilcoxon rank sum test. The level of statistical significance of cell type-specific transcriptome changes was set to adjusted P & LT; 0.1 and log2 multiples change ≥0.25 or ≤ −0.25.

#### 2.2.4. Gene-set enrichment analysis (GSEA)

Gene-set enrichment analysis was applied to identify *a priori*-defined gene sets that show statistically significant differences between two given clusters. The expression file was set as input, and gene sets of KEGG pathways and Gene Ontology (GO) were implied, which were collected in Molecular Signatures Database (MSigDB) (Subramanian et al., 2005; Zhong et al., 2018).

### 2.3. GO and KEGG signaling pathway enrichment analysis

In this study, DEGs were all mapped to the GO terms in the Gene Ontology database,^3^ and the number of genes were calculated for each term. Pathway-based analysis was used to characterize the biological functions of the genes. Pathway enrichment analysis identified significant signal transduction pathways in the KEGG database.^4^ In GO and KEGG enrichment analysis, R software version 3.8.1,^5^ and multiple R packages, such as *clusterProfiler, org.Hs.eg.db, enrichplot* and *ggplot2*, were used to generate the bars, bubble maps, and signaling pathway maps.

### 2.4. PPI network analysis

The STRING database^6^ was used for DEG-associated protein interaction analysis and production of PPI networks. Cytoscape 3.8.0 was used^7^ to construct the cell differential expression network.

### 2.5. Participant information, inclusion criteria and ELISA assay

Participant in the age range of 20–90 years were recruited. All of them met the following inclusion criteria to be considered in this study: no head injury at time of death, lack of developmental disorder, no recent cerebral stroke, no history of other psychiatric or neurological disorders, and no history of intravenous or polydrug abuse. Twenty healthy adult volunteers aged between 20 and 27 years were selected as the adult group, and 20 elderly volunteers aged between 64 and 84 years were arranged into the elderly group. All volunteers were informed of the purpose of the study and signed informed consent forms. Blood samples collected from participants were coagulated and centrifuged. Afterward, plasma was collected and stored in Eppendorf tubes until use. The levels of CA10, DSCAM, GPC5, IL1RAPL2, CNTN2 and SPOCK3 proteins in plasma samples were detected by ELISA kit. The operation and detection were performed strictly according to the kit instructions as previously described (Yu et al., 2022). A spectrophotometer (Thermo Fisher Scientific, Vantaa, Finland) was applied to measure the absorbance [optical density (OD) value] of each sample at a wavelength of 450 nm for 15 min. Finally, the linear regression equation of the standard curve was calculated by using the concentration and OD of the standard product. Then, the concentration of each protein in the plasma was calculated.

### 2.6. Statistical analysis and data visualization

snRNA-seq data were analyzed using the Seurat package in R. *P*-values were adjusted based on Bonferroni correction. For ELISA assays, differences between aging and adult were analyzed using Student’s *t*-test (SPSS v26.0, IBM, USA). *P* < 0.05 was considered statistically significant. We visualized the data using Cytoscape (version 3.8.0) and ggplot2 package in R.

## 3. Results

### 3.1. Cell-specific transcriptional profiles of the prefrontal cortex of human brain aging

**FIGURE 1:**
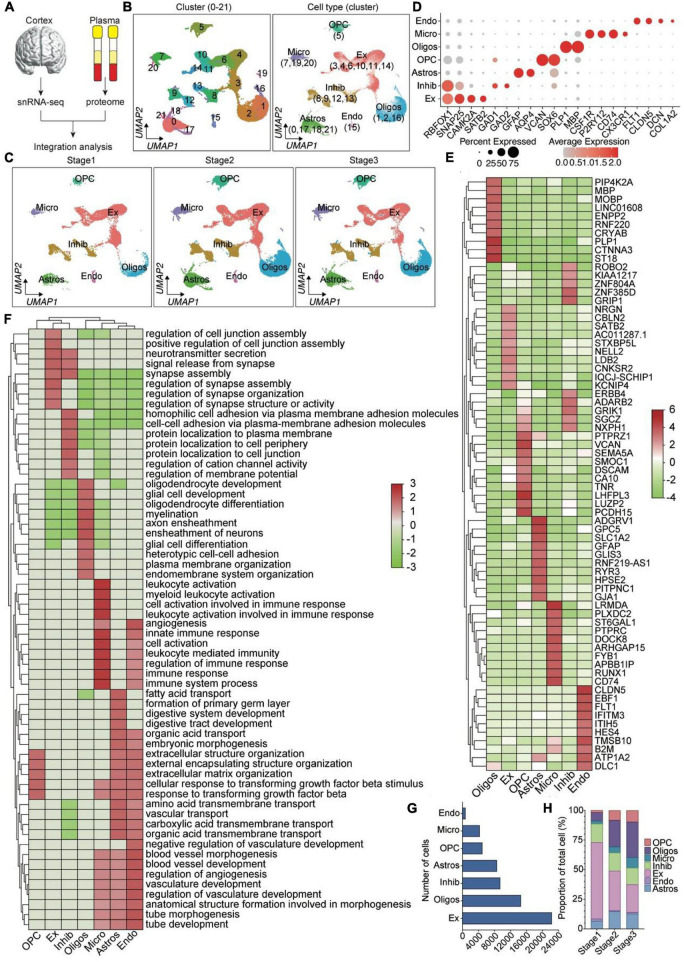
Integration analysis of snRNA-seq data for prefrontal cortex during aging. **(A)** Integrated analysis workflow. **(B,C)** Unbiased recognition of cell-type heterogeneity in the human prefrontal cortex. **(B)** The UMAP plot shows distribution of different cluster (left) and cell types (right) in the PFC. **(C)** The distribution of different cell types in the stage1 (left), stage2 (center) and stage3 (right) PFC. **(D)** Bubble dot plots of the top cluster-specific marker genes. The size of the dot indicates expression percentage and the darkness of the color indicates average expression. **(E)** The heatmap of top 10 DEGs in each cell types. **(F)** GSEA analysis of seven cell types. **(G)** Total captured cell number for each cell type. **(H)** The bar plots show the proportion of the seven cell types found in the PFC samples during aging. Oligos, oligodendrocyte; Ex, excitatory; OPC, oligodendrocyte progenitor cells; Astros, astrocytes; Micro, microglia; Inhib, inhibitory; Endo, endotheliocyte.

To investigate how molecular and cellular features are altered during brain aging in healthy individuals, we integrated the data from two snRNA-seq datasets (Grubman et al., 2019; Brenner et al., 2020; Lau et al., 2020) using the method of Stuart et al. (2019) (Seurat3). Seurat3 uses canonical correlation analysis (CCA) and mutual nearest neighbor (MNN) for data integration, which can effectively remove batch effect between samples. The analysis flow is shown in Figure 1A, and two datasets containing snRNA-seq data of prefrontal tissues from 14 normal healthy individuals across young adults to nonagenarians were analyzed. These samples were divided into three groups according to age, namely stage1 (adult, age 53.5 ± 6.4), stage2 (early-aging, age 75.0 ± 4.5), and stage3 (late-aging, age 89.7 ± 3.4) (Supplementary Table 1). After standardized integration and quality control, a total of 65,064 cells (stage1: 10343, stage2: 28897, stage3: 25824) were obtained for subsequent downstream processing (Supplementary Table 2).

To construct taxonomic maps of cell populations, all data were subjected to principal component analysis (PCA) and uniform manifold approximation and projection (UMAP) clustering analysis and visualized, which yielded 22 unique cell clusters (Figures 1B, C). Comparing differential genes across all cell types, the specific genes expressed by each cell type were revealed to identify cell phenotype (Figures 1D, E). Seven major cell types were classified based on their respective transcriptomic expression profiles and the previously reported cell type marker genes (Mathys et al., 2019; Lau et al., 2020): Astrocytes (Astros, C0, C17, C18, and C21; marked by AQP4/GFAP, 13.3% of the total), endotheliocytes (Endo, C15; EBF1/RGS5, 1.2%), excitatory neuron (Ex, C3, C4, C6, C10, C11, and C14; CAMK2A/SATB2, 34.3%), inhibitory neuron (Inhib, C8, C9, C12, and C13; GAD1 and GAD2, 14.5%), microglia (Micro, C7, C19 and C20; PTPRC/CSF1R, 6.6%), oligodendrocyte (Oligos, C1, C2 and C16; marked by MOG/PLP1/ST18, 22.7%), and OPC (OPC, C5; VCAN/PDGFRA, 7.6%) (Figures 1B–G and Supplementary Tables 2, 3). GSEA of these cell-type-specific marker genes demonstrated functional characteristics of the corresponding cell type (Figure 1F). For example, “synapse assembly and organization” was enriched for the top marker genes (log2FC > 0.25) for Ex and Inhib, “myelination and glia cell development” for Oligos, “leukocyte activation” for microglia, “angiogenesis” for End, etc (Figure 1F). In order to investigate how molecular and cellular characteristics of prefrontal cortex are altered during aging in healthy individuals, we compared the proportion of different cell types across three stages. We found that the proportion of Ex was sharply decreased with age, while the OPC, Oligos, Micro was increased (Figure 1H and Supplementary Table 3). The proportion of Astros was increased in the early-aging (Figure 1H and Supplementary Table 3). Then, To identify overall transcriptomic changes for each cell type across three stage, we compared the transcriptomic profiles of individual cell types across three age groups. A total of 93 differentially expressed genes (DEGs) were identified for all cell type during aging. Among them, genes upregulated with age include 5 astrocytes, 13 endotheliocytes, 5 microglia; excitatory neurons, inhibitory neurons, oligodendrocytes, and OPC are not significantly upregulated with age (Supplementary Table 4). Genes down-regulated with age include 2 astrocytes, 6 endotheliocytes, 16 excitatory neurons, 23 inhibitory neurons, 11 microglia, 6 oligodendrocytes, 6 oligodendrocyte precursors (Supplementary Table 4).

### 3.2. Acquisition of differential genes/proteins between prefrontal cortex and plasma associated with aging

In order to investigate how molecular and cellular characteristics of prefrontal cortex are altered during aging in healthy individuals, we compared the transcriptomic profiles of individual cell types across three age groups. A total of 870 DEGs were identified for each cell type during aging (Supplementary Tables 5–18). Then, we compared levels of gene expression in cells across all stage by cell type, and identified 93 unique DEGs that is constantly changing in all stages (Supplementary Table 4).

**FIGURE 2:**
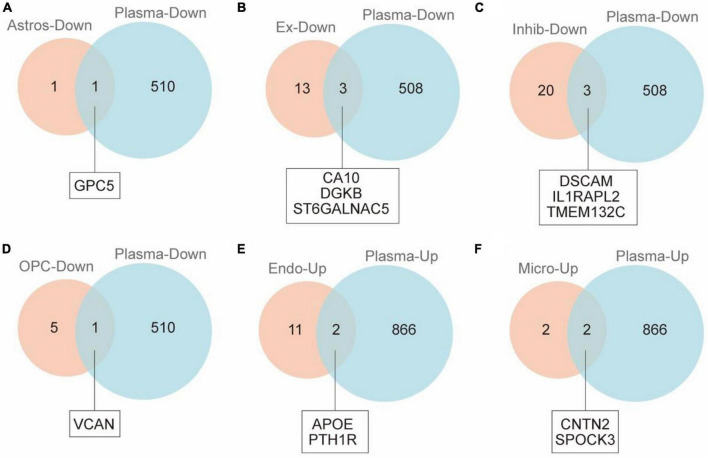
VENNY map of cerebral cortex and plasma proteins in naturally aging patients. **(A)** Venn diagram shows overlap of DEGs of astrocytes and DEPs of plasma. **(B)** Venn diagram shows overlap of DEGs of excitatory neurons and DEPs of plasma. **(C)** Venn diagram shows overlap of DEGs of inhibitory neurons and DEPs of plasma. **(D)** Venn diagram shows overlap of DEGs of OPCs and DEPs of plasma. **(E)** Venn diagram shows overlap of DEGs of endotheliocyte and DEPs of plasma. **(F)** Venn diagram shows overlap of DEGs of microglia and DEPs of plasma.

In order to identify the cell-specific plasma markers of brain aging, we further collected and analyzed the plasma proteomics data from 2,925 plasma proteins across 4,263 young adults to nonagenarians (18–95 years old). There were 1,376 differential proteins in the plasma that changed significantly with age (Figure 2). By integrated analysis of snRNA-seq data and proteomic data, we found 12 differentially expressed genes/proteins (GPC5, CA10, DGKB, ST6GALNAC5, DSCAM, IL1RAPL2, TMEM132C, VCAN, APOE, PYH1R, CNTN2, SPOCK3, Table 1) from astrocytes (Figure 2A), excitatory neurons (Figure 2B), inhibitory neurons (Figure 2C), oligodendrocyte progenitors (Figure 2D), endothelial cells (Figure 2E), and microglia (Figure 2F).

**TABLE 1:** Differential gene expression information.

Source	Gene name	Protein name	Gene expression
Astrocyte	GPC5	Glypican proteoglycan 5	Down
Excitatory neuron	CA10	Carbonic anhydrase 10	Down
Excitatory neuron	DGKB	Diacylglycerol kinase beta	Down
Excitatory neuron	ST6GALNAC5	ST6 N-acetylgalactosaminide alpha-2,6-sialyltransferase 5	Down
Inhibitory neuron	DSCAM	DS cell adhesion molecule	Down
Inhibitory neuron	IL1RAPL2	Interleukin 1 receptor accessory protein like 2	Down
Inhibitory neuron	TMEM132C	Transmembrane protein 132C	Down
OPC	VCAN	Versican	Down
Endotheliocyte	APOE	Apolipoprotein E	Up
Endotheliocyte	PTH1R	Parathyroid hormone 1 receptor	Up
Microglia	CNTN2	Contactin 2	Up
Microglia	SPOCK3	SPARC (osteonectin), Cwcv and kazal like domains proteoglycan 3	Up

### 3.3. GO, KEGG, and PPI analysis of differential genes

**FIGURE 3:**
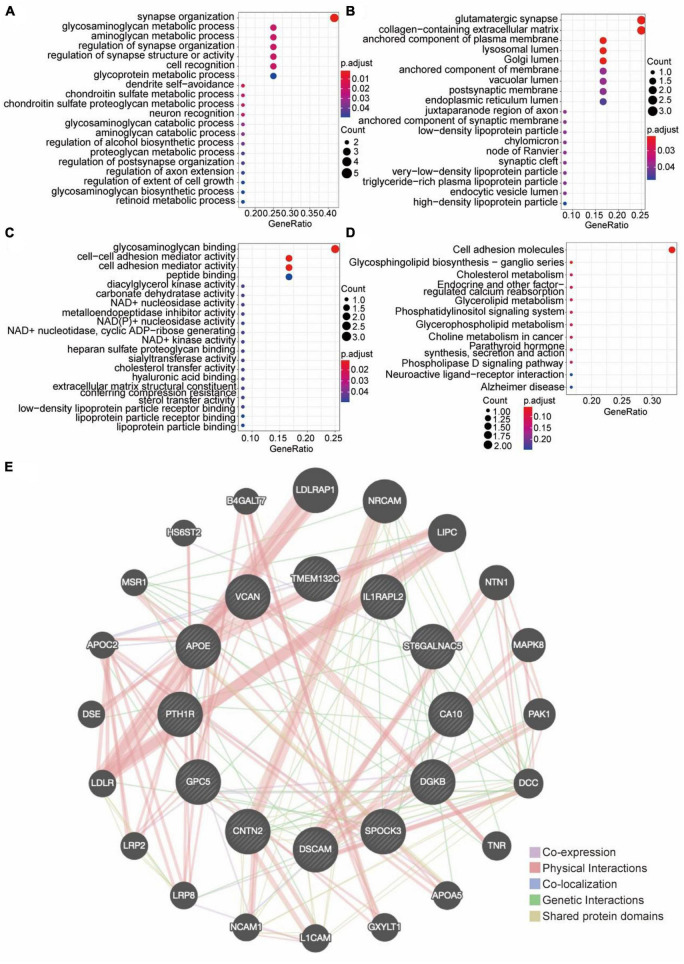
The functional enrichment analysis. **(A–C)** GO functional enrichment analysis. **(A)** The top 20 GO enrichments in BP. **(B)** The top 20 GO enrichments in CC. **(C)** The top 20 GO enrichments in MF. Each node signaled a GO term, and its size represented the gene number. The color indicates the *p*-value. **(D)** KEGG enrichment analysis and pathway mapping. **(E)** Differential Gene PPI Network Analysis diagram. Each circle in the diagram represents a differentially expressed gene. The larger the circle, the higher the expression of DEG. The line connecting the DEGs represents the correlation between them. The thicker the connection line, the stronger the correlation between DEGs.

In order to elucidate the biological functions of these 12 molecules, GO functional enrichment analysis and KEGG signaling pathway analysis were performed (Figure 3). The 12 genes/proteins were mainly enriched in these biological processes (BP): synapse organization and aminoglycan metabolic process (Figure 3A), molecular function (MF): glycosaminoglycan binding and cell-cell adhesion (Figure 3B), cell component (CC): anchored component of Plasma membrane, Glutamatergic synapse, Lysosomal Lumen (Figure 3C). KEGG analysis showed that these crucial genes were mainly enriched in cell adhesion molecules (Figure 3D). In order to identify whether there was a relationship among these 12 molecules, the protein-protein interaction analysis was performed and the PPI network was obtained (Figure 3E). The PPI network showed that there was extensive direct and indirect communication, physical and genetic interactions among these 12 molecules.

## 4. Verification of the brain aging markers

**FIGURE 4:**
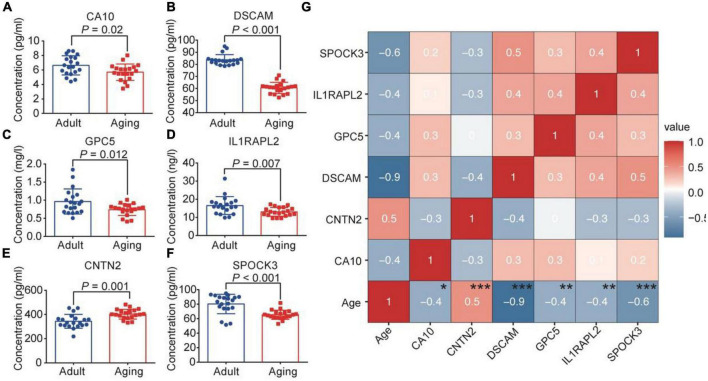
ELISA test verified the expression of brain aging markers. Plasma expression level of CA10 **(A)**, DSCAM **(B)**, GPC5 **(C)**, IL1RAPL2 **(D)**, CNTN2 **(E)**, SPOCK3 **(F)** in young and elderly groups. **(G)** Correlation analysis of the six molecules with age. *p < 0.05, **p < 0.01, ***p < 0.001.

To verify the results obtained above, we further collected plasma from 20 young individuals and 20 elderly individuals (Supplementary Table 19). By consulting relevant literature, 6 genes related to neurodevelopmental and neurological diseases (GPC5, CA10, DSCAM, IL1RAPL2, CNTN2, and SPOCK3) were verified by ELISA method (Supplementary Table 20). The ELISA results demonstrated that compared with the adult group, GPC5, CA10, DSCAM, IL1RAPL2, and SPOCK3 were down-regulated in the aging group (Figures 4A–D, F). CNTN2 showed an up-regulation trend with significant differences (Figure 4E). The expression trends of GPC5, CA10, DSCAM, IL1RAPL2, and CNTN2 were consistent with snRNA-Seq and proteomic outcomes. Moreover, correlation analysis of these genes with age revealed that there are significantly age-related change trends for these molecules (Figure 4G).

## 5. Discussion

It is well known that the degeneration process of various physiological functions caused by aging is particularly marked by the decline in cognitive ability of the brain. Aging is a major risk factor for human neurodegenerative disorders, such as Alzheimer’s disease (AD), which is rapidly rising in prevalence with age and has become the most pressing challenge to be tackled (Zhang et al., 2021). The brain aging process is mainly reflected in the shrinkage of the whole brain, the reduction of gray and white matter, and the enlargement of the ventricle (Drayer, 1988). Magnetic resonance imaging (MRI) studies have shown that age-related gray matter loss is most prominent in the temporal and frontal lobes (Jack et al., 1997). Histological analysis has shown that brain atrophy is the result of a combination of physiological degeneration such as dendritic degeneration and neuronal death (Dumitriu et al., 2010). Thus, the degree of brain atrophy during aging can broadly predict the onset of cognitive impairment and the degree of cognitive decline (Jack et al., 2005). It has also been suggested that although there are individual differences in the rate of brain atrophy during aging, brain imaging data can still be used to initially determine the “biological age” of a person’s brain (Cole and Franke, 2017), but this method has limitations for primary clinical screening. The resolution and accuracy of MRI in the prediction of brain aging remains modest, failing to predict the changes of brain structure or function more accurately on early onset Previous exploratory analysis of the brain at the cellular and molecular levels has revealed many distinct features of brain aging, including mitochondrial dysfunction, intracellular accumulation of oxidatively damaged proteins, nucleic acids and lipids, dysregulated energy metabolism, impaired cellular “waste disposal” mechanisms autophagy lysosome and proteasome function), impaired adaptive stress response signaling, impaired DNA repair, abnormal neural network activity, dysregulated neuronal Ca2+ processing, stem cell depletion and neuroinflammation, etc (Mattson, 2000; Shimura et al., 2001; López-Otín et al., 2013; Chow and Herrup, 2015; Cohen et al., 2015). However, it is still difficult to establish and widely promote the above-mentioned aging indicators and methods in clinical practice. So it is especially important to find and establish a new authoritative screening index and method related to brain aging. Recent studies utilized single-nucleus transcriptome analysis to investigate the transcriptomic changes in aging brains and have revealed molecular alterations at the single-cell level using readily available frozen brain tissues (Lau et al., 2020). However, snRNA-seq has not been applied to explore the cellular and molecular alterations of brain aging in the human. At present study, we first performed snRNA-seq analyses of the post-mortem human PFC and generated profiles of 65,064 nuclei from 14 subjects across adult (44–58 years), early-aging (69–79 years), and late-aging (85–94 years) stages. A total of 93 cell-specific genes were identified to be significantly associated with age. Combined with proteomics data, we screened 12 differential genes/proteins (GPC5, CA10, DGKB, ST6GALNAC5, DSCAM, IL1RAPL2, TMEM132C, VCAN, APOE, PTH1R, CNTN2, SPOCK3) associated with aging. Among these genes, we found decreased expression of GPC5 derived from astrocytes, VCAN derived from OPC, DSCAM, IL1RAPL2, TMEM132C derived from inhibitory neurons and CA10, DGKB, ST6GALNAC5 derived from excitatory neurons. Meanwhile, the expression of APOE and PTH1R derived from endothelial cells, and CNTN2 and SPOCK3 derived from microglia were increased. Finally, six genes/proteins related to neurodevelopmental and neurological diseases (GPC5, CA10, DSCAM, IL1RAPL2, CNTN2, and SPOCK3) were verified by ELISA.

In the Elisa sample collection process, we selected samples in the age range of 18–95 years. The age span of the young group increased relative to the age of the sample in the snRNA-seq analysis, and we used this to more broadly verify the differences and trends of the predicted proteins between the young and old groups. Referring to the sample size verified by Elisa experimental data in previous articles of this kind, we selected 40 subjects of different ages for sampling and subsequent Elisa verification and analysis.

Glypican proteoglycan 5 (GPC5) is a class of heparan sulfate proteoglycans bound to the outer surface of the plasma membrane, and all members of its family are expressed during the development of the nervous system. This protein plays an important role in the control of cell division and growth regulation involved in brain patterning, synapse formation, axon regeneration and guidance and has also been found in a dense network of active MS plaques (cerebral atherosclerotic plaques) and members of this family may be involved in the isolation of pro-inflammatory chemokines (Kenny et al., 2017). Luxardi et al. (2007) found that GPC5 plays an important regulatory role in cell signaling during embryogenesis, while Gpc5 is expressed in the ventral brain when neurogenesis begins. The presence of this protein in human and mouse brains involves in brain development and neural function, including neuron growth and repair (Veugelers et al., 1997; Taniuchi et al., 2002a). In adults, GPC5 is mainly expressed in neurons of brain tissue (Kenny et al., 2017) and it interacts with growth factors, chemokines and extracellular matrix proteins. At present study, we found that the GPC5 expression of astrocytes was down-regulated with age. In combination with the ELISA experiment, GPC5 showed a down-regulation trend in the elderly group, corresponding to the snRNA-seq and plasma results. Recent work has shown that in addition to being supportive cells, astrocytes are crucial in controlling vascular blood flow, axon guidance, synaptic formation, regulating synaptic transmission and neural circuits, which are essential for the development and function of the central nervous system (Zhang et al., 2016; Chai et al., 2017; Verkhratsky and Nedergaard, 2018; Huang et al., 2020). Although astrocytes promote neuron survival and synaptic formation, their ability to promote synaptic formation tends to decline with age (Zhang et al., 2016). Moreover, GPC5 can stimulate the Shh signaling pathway to promote neuron growth and synaptic formation (Li et al., 2011). Therefore, the down-regulation of GPC5 in astrocytes may indicate the downregulation of synaptic formation function in the brain, thus causing cognitive impairment.

Carbonic anhydrases (CA) are present in many organisms where they are involved in several important biological processes. Romeo et al. (2009) showed that this protein and its family members are mainly expressed on neurons, especially on neuronal axons. A research report related to glypican genes in neural progenitor cells and differentiated neurons also pointed out that the expression of CA was found in some mouse and human tissues, and immunohistochemical results showed that their expression was mainly concentrated in brain tissue compared to other organ (Taniuchi et al., 2002a,b). Some studies have also shown that CA10 mRNA expression levels are high in the cerebellum, frontal cortex and parietal cortex, low in the midbrain, and extremely low in the eye (Aspatwar et al., 2010). Analysis of genome-wide genotyping BeadChip assays in clinically relevant patients revealed that when CA10 and its related genes involved in brain development and neurological processes are absent, patients exhibit mild intellectual disability, growth retardation, poor weight gain, microcephaly, long face, large beaked nose, thick lower lip, micrognathia and other dysmorphic features (Preiksaitiene et al., 2012). Consistently, we found that the CA10 expression levels in excitatory neuron were down-regulated with age and is reflected in plasma levels. These results suggested that the decreased of CA10 in aging brain may lead to a series of cognitive decline problems.

DSCAM is a member of the cell adhesion molecule (Ig-CAM) immunoglobulin superfamily and is involved in the development of the human central and peripheral nervous system. DSCAM is expressed in the nervous system of invertebrates and vertebrates, as well as a candidate gene for Down syndrome and congenital heart disease (DSCHD) (Barlow et al., 2001; Boulanger, 2009). DSCAM is involved in cell migration, synaptic targeting, axon guidance, dendrites and cell tiling, axon fascicles and branching, programmed cell death and synaptogenesis in the nervous system, all of which can influence the establishment of motor circuits during development and the effects are maintained into adulthood (Garrett et al., 2012). There is growing evidence that DSCAM expressed in vertebrates also plays an important role in axonal growth, fasciculation and branching, dendritic arborization, mosaic spacing of cells, and synaptogenesis (Garrett et al., 2012; Montesinos, 2014, 2017; Mitsogiannis et al., 2020), and that this protein promotes pyramidal neuron morphogenesis by regulating dendritic arborization and spine formation during cortical circuit development (Agarwala et al., 2001; Maynard and Stein, 2012). So far, we experimentally showed that DSCAM was significantly down-regulated in the elderly group. Combining previous research and analysis by other scholars, when it was decreased in adult brain, the axonal and dendritic developmental processes and intercellular interactions of various neuronal cells are also adversely affected, leading to a series of neurodegenerative diseases and behaviors (Li et al., 2023). Accordingly, the down-regulation of this protein may also predict brain aging and cognitive disorder.

IL1RAPL2 is a member of the interleukin 1 receptor family and located on the X chromosome in a region associated with X-linked non-syndromic cognitive impairment. Ferrante et al. (2001) showed that IL1RAPL2 is specifically expressed in the nervous system from embryonic day 12.5 through *in situ* expression studies on RNA of mouse embryonic tissue sections at different developmental stages. IL1RAPL2 has a restricted and specific expression in the brain during development, which may point to a precise role of this gene in the development or function of the nervous system (Ferrante et al., 2001). Because of its specific expression pattern, it is restricted to structures related to cognitive function, such as the PFC and hippocampus, which is also consistent with our experimental results that the gene is down-regulated in the old group. Therefore, it also suggests that IL1RAPL2 could be used as a candidate protein for brain aging to predict brain aging.

Current research on specific protein markers for neurodegenerative diseases has evaluated 30 highly specific proteins associated with the brain as candidate biomarkers for the diagnosis of AD, Among these proteins, CNTN2 may also be indicators of disease progression, showing weak to moderate correlation with cognitive tests, which contain the CNTN2 protein of our interest (Begcevic et al., 2018). Some scholars believe that CNTN2 is expressed in neurons and oligodendrocytes and is highly regulated in developing neurons, especially in axonal growth and guidance and neuronal migration (Huang et al., 2014). On the other hand, CNTN2 expressed by oligodendrocytes interferes with the process of myelination, and its ablation leads to hypomyelination. Wang et al. (2018) and Zou et al. (2020) demonstrated through a series of experiments that CNTN2 overexpression or knockdown could reverse the effects of miR-34a upregulation or downregulation on proliferation and migration of Schwann cells, respectively. Combined with our experimental results, CNTN2 showed an up-regulation trend in the elderly group, then we can speculate that this protein can also be one of the candidate blood markers of brain aging. However, the age-related CNTN2 we found was mainly derived from microglia, so the dysfunction caused by the rise of CNTN2 in microglia require further investigation. SPOCK3 is a heparan sulfate proteoglycan, also known as testosterone-3, expressed in the nervous system. Some scholars have also stated that SPOCK1 is a member of the Spock family, and Spock1 inhibits the attachment to the substrate and the neurite growth of neuronal cells, so SPOCK3 is also considered to be related to neuron development (Yamamoto et al., 2014). At present study, SPOCK3 protein showed an increasing trend from the snRNA-seq and plasma proteomics, but the experimental results were contrary to it. This situation may be related to the multiple and complex regulatory roles of spock3 protein.

In conclusion, this article presents a validation and analysis of biomarkers of brain aging in blood. The biomarkers of brain aging in blood were screened by the analysis of human cerebral cortex scRNA-seq data and plasma proteome. After validation of the predicted and screened candidate proteins by Elisa method using peripheral blood plasma from young and old groups, GPC5, CA10, DSCAM, IL1RAPL2, CNTN2 have certain correlation with age. These proteins can be combined with imaging detection methods to provide certain reference value for the prevention and diagnosis of brain aging, and also provide new ideas and directions for future research on brain aging related topics.

## Data availability statement

The snRNA-seq data after integration and code reported in this manuscript have been deposited in the figshare (https://figshare.com/), under doi: 10.6084/m9.figshare.22634395.

## Ethics statement

The studies involving human participants were reviewed and approved by the Medical Ethics Committee of Kunming Medical University (approval number: KMMU2022MEC092). The patients/participants provided their written informed consent to participate in this study. Written informed consent was obtained from the individual(s) for the publication of any potentially identifiable images or data included in this article.

## Author contributions

R-ZN and W-QF analyzed the data and drafted the manuscript. L-LS, Q-SY, and Q-MQ discussed and revised the manuscript. R-ZN and JL designed the research and collected the information. All authors read and approved the final manuscript.

## Footnotes

1
https://www.ncbi.nlm.nih.gov/geo/

2
https://twc-stanford.shinyapps.io/aging_plasma_proteome/

3
http://geneontology.org/

4
http://www.genome.jp/kegg/

5
http://www.r-project.org

6
https://www.string-db.org/

7
https://cytoscape.org/
